# Chronic Progressive External Ophthalmoplegia: A Case Report

**DOI:** 10.7759/cureus.77149

**Published:** 2025-01-08

**Authors:** Rajiv V Seemongal-Dass, Durell J Gracen, Robin R Seemongal-Dass, Bernard Chang

**Affiliations:** 1 Ophthalmology, Eyenet Ltd, Chaguanas, TTO; 2 Department of Ophthalmology, Leeds Teaching Hospitals NHS Trust, Leeds, GBR

**Keywords:** chronic progressive external ophthalmoplegia, cpeo, mitochondrial disease, ophthalmoplegia, ptosis

## Abstract

Chronic progressive external ophthalmoplegia (CPEO) is a rare mitochondrial disorder characterized by bilateral, slowly progressive ptosis and paralysis of the extraocular muscles. We present the case of a 61-year-old female with a 36-year history of bilateral ptosis and limited eye movements without diplopia. No family history of CPEO or other mitochondrial disorders was reported. To the best of our knowledge, this is the first documented case of CPEO in Trinidad and Tobago.

## Introduction

Chronic progressive external ophthalmoplegia (CPEO) is a rare mitochondrial disorder within the spectrum of mitochondrial DNA deletion syndromes. It typically presents with slowly progressive bilateral ptosis and ophthalmoplegia; diplopia is usually absent, and pupillary function is preserved. Onset can depend on the specific form of CPEO and can occur as early as adolescence or young adulthood, though it may vary [1].

Most cases are sporadic, though familial cases may follow autosomal dominant, autosomal recessive, or maternal inheritance patterns. Muscle biopsy is the gold standard for diagnosis, revealing characteristic cytochrome C-oxidase deficiencies and ragged-red fibers. Orbital imaging typically shows atrophy of the extraocular muscles [2].

## Case presentation

A 61-year-old female of African descent from Trinidad with known CPEO presented with progressive bilateral ptosis and limited extraocular movement, which she had experienced for 36 years. There was no diplopia, nystagmus, or family history of mitochondrial disease. Her past ocular and surgical history included bilateral ptosis correction surgery 17 years earlier for which she was diagnosed with CPEO one year after. She also underwent a total abdominal hysterectomy with ovarian conservation. Her past medical history includes hypertension for two years.

She was highly myopic. Her ophthalmic assessment revealed a visual acuity of 6/48-1 OD and 6/15-2 OS, improving to 6/24+2 OD and 6/15-2 OS with pinhole correction and N9 for near vision. Intraocular pressures were 19 mmHg OD and 21 mmHg OS. Pachymetry revealed central corneal thicknesses of 506 μm OD and 518 μm OS and axial lengths of 28.11 mm OD and 26.95 mm OS. Subjective refraction was -10.25/-3.25 x158 OD and -7.75/-0.25 x127 OS.

**Figure 1:**
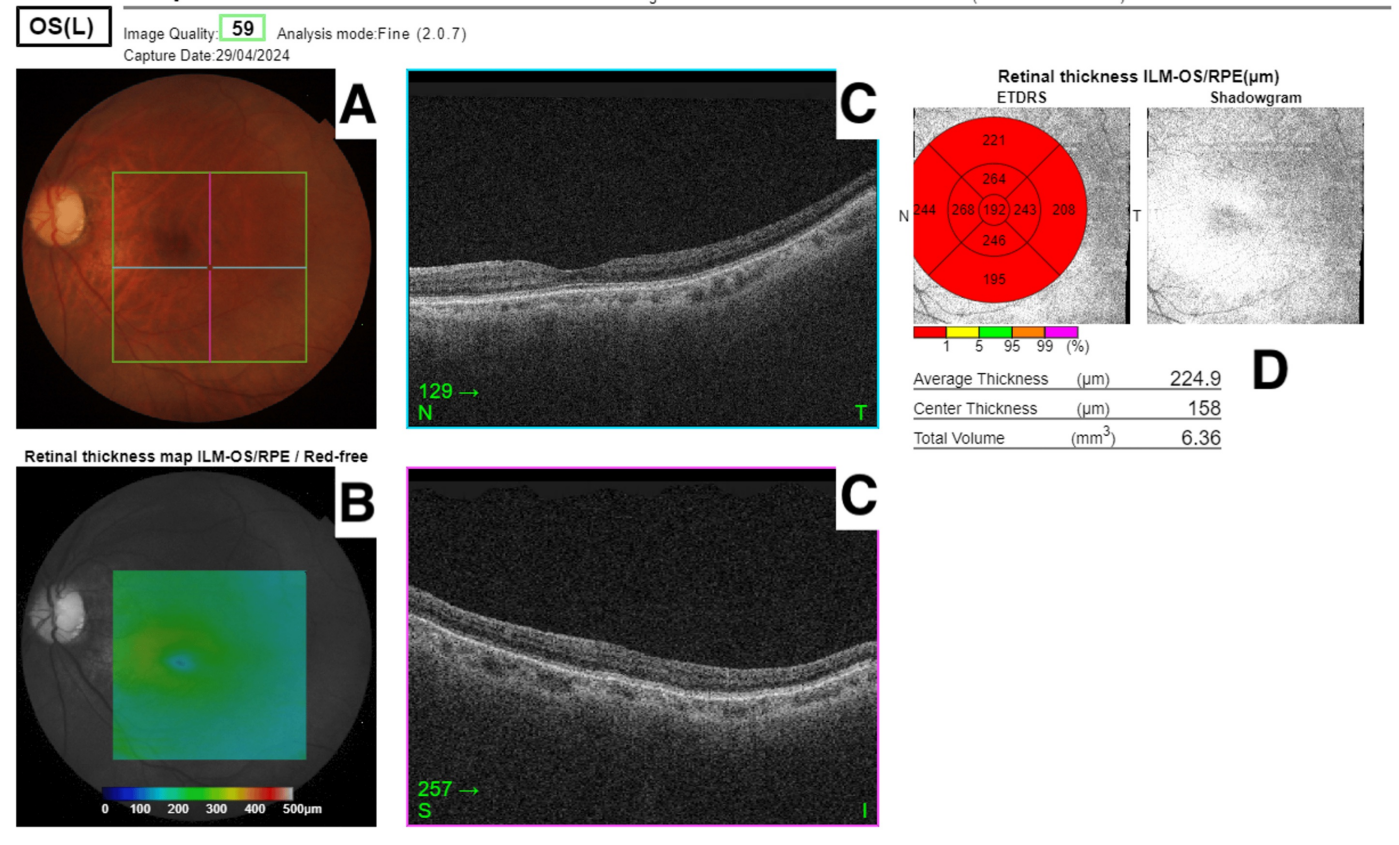
Left optical coherence tomography of the macula showing myopic changes of the fundus with no pigmentary retinopathy. A: left fundus image B: left retinal thickness map (red-free) C: left retinal layers - plane T (top surface) and plane S (bottom surface) D: left retinal thickness map with ETDRS grid overlay Abbreviations: ETDRS, early treatment diabetic retinopathy study

**Figure 2:**
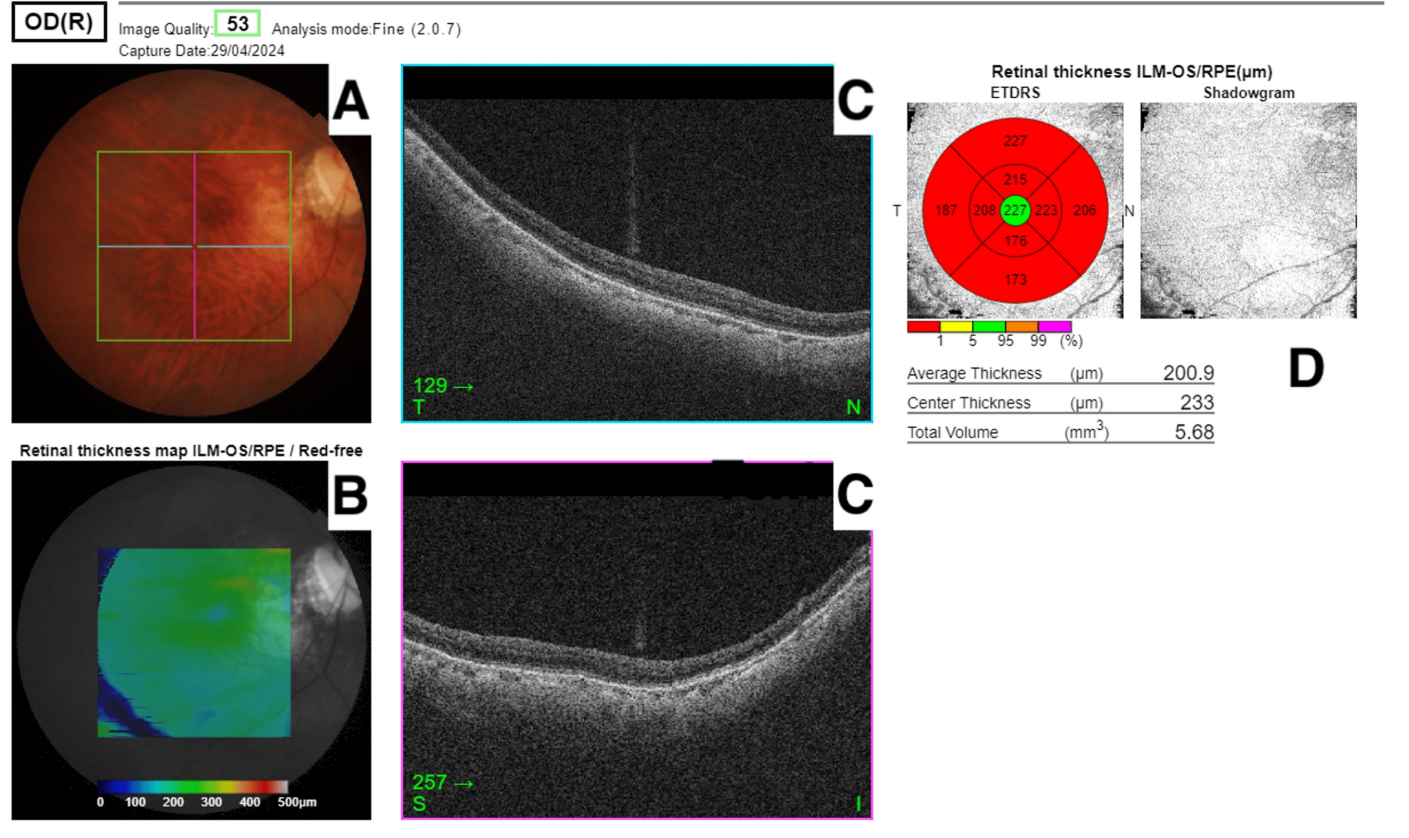
Right OCT of the macula showing myopic changes of the fundus with no pigmentary retinopathy A: right fundus image B: right retinal thickness map (red-free) C: right retinal layers - plane T (top surface) and plane S (bottom surface) D: Right retinal thickness map with ETDRS grid overlay Abbreviations: OCT, optical coherence tomography; ETDRS, early treatment diabetic retinopathy study

On examination, she exhibited severe bilateral ptosis and severely limited extraocular movements in all directions. Pupillary reactions were normal, with no relative afferent pupillary defect. Slit-lamp examination and indirect ophthalmoscopy revealed no evidence of optic atrophy or retinal pigmentary degeneration. Optical coherence tomography (OCT) revealed changes consistent with high myopia (Figures 1-2).

Teleconsultation was arranged with an oculoplastic surgeon based in the United Kingdom and it was recommended that the patient undergo cataract surgery before ptosis correction. She subsequently underwent successful bilateral cataract surgeries, with phacoemulsification and intraocular lens implantation (8.0 D OD, 9.0 D OS monofocal). Despite improvements in visual acuity (unaided VA of 6/18 OD and 6/9-1 OS), her ptosis worsened postoperatively, and she developed moderate exotropia without diplopia.

**Figure 3:**
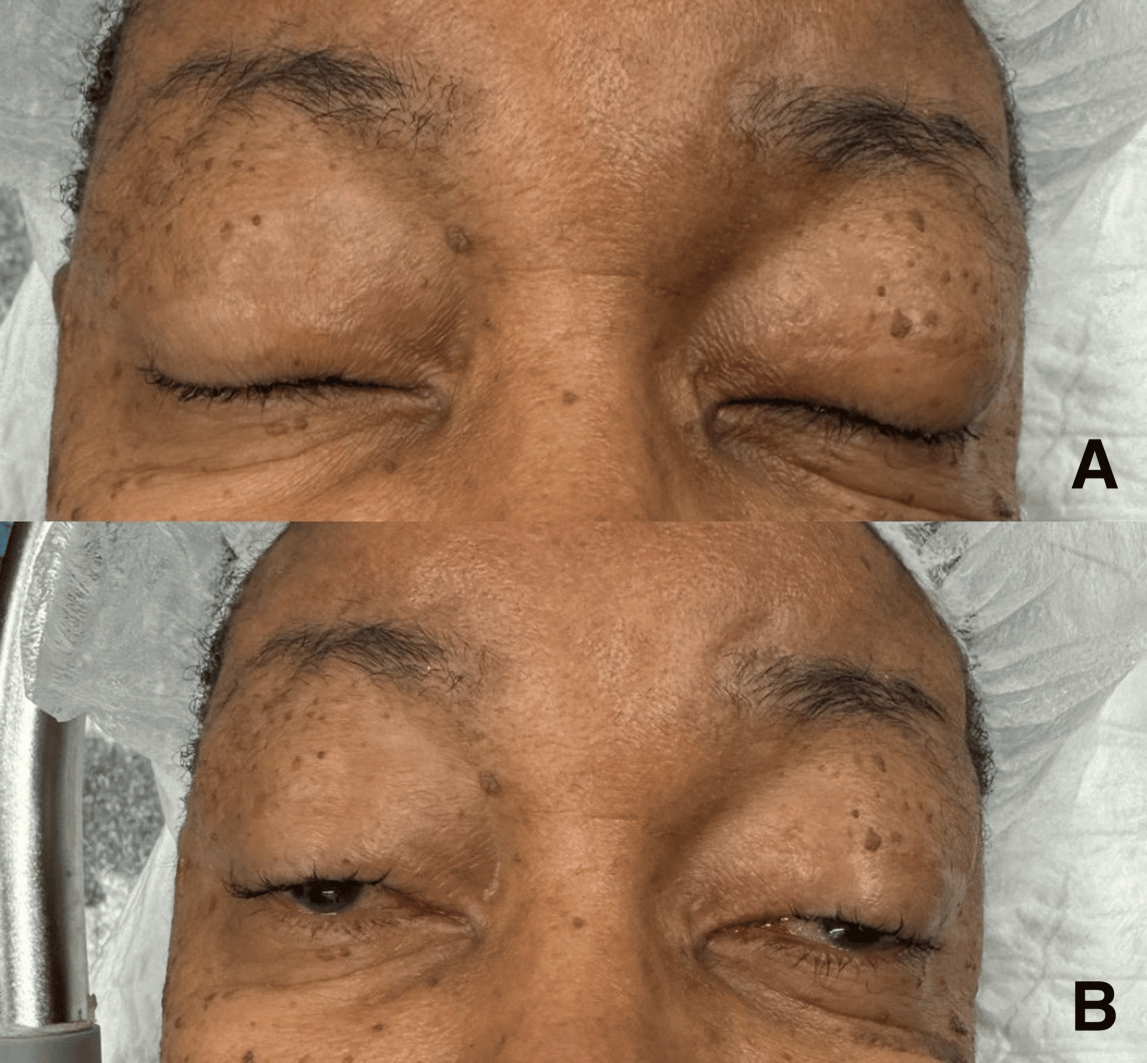
Maximal Eyelid Opening and Closing Prior to Ptosis Repair. Also seen are ophthalmoplegia, loss of upper eyelid crease and exotropia. A: Eyelid fully closed B: Eyelid fully opened

Bilateral ptosis surgery with brow suspension was performed under general anesthesia using the Crawford technique with silicone slings [3]. In CPEO, progressive muscle weakness leads to ptosis (as seen in Figure 3A-3B) and limited eye movement, necessitating the use of this technique to bypass the weakened levator muscles.

**Figure 4:**
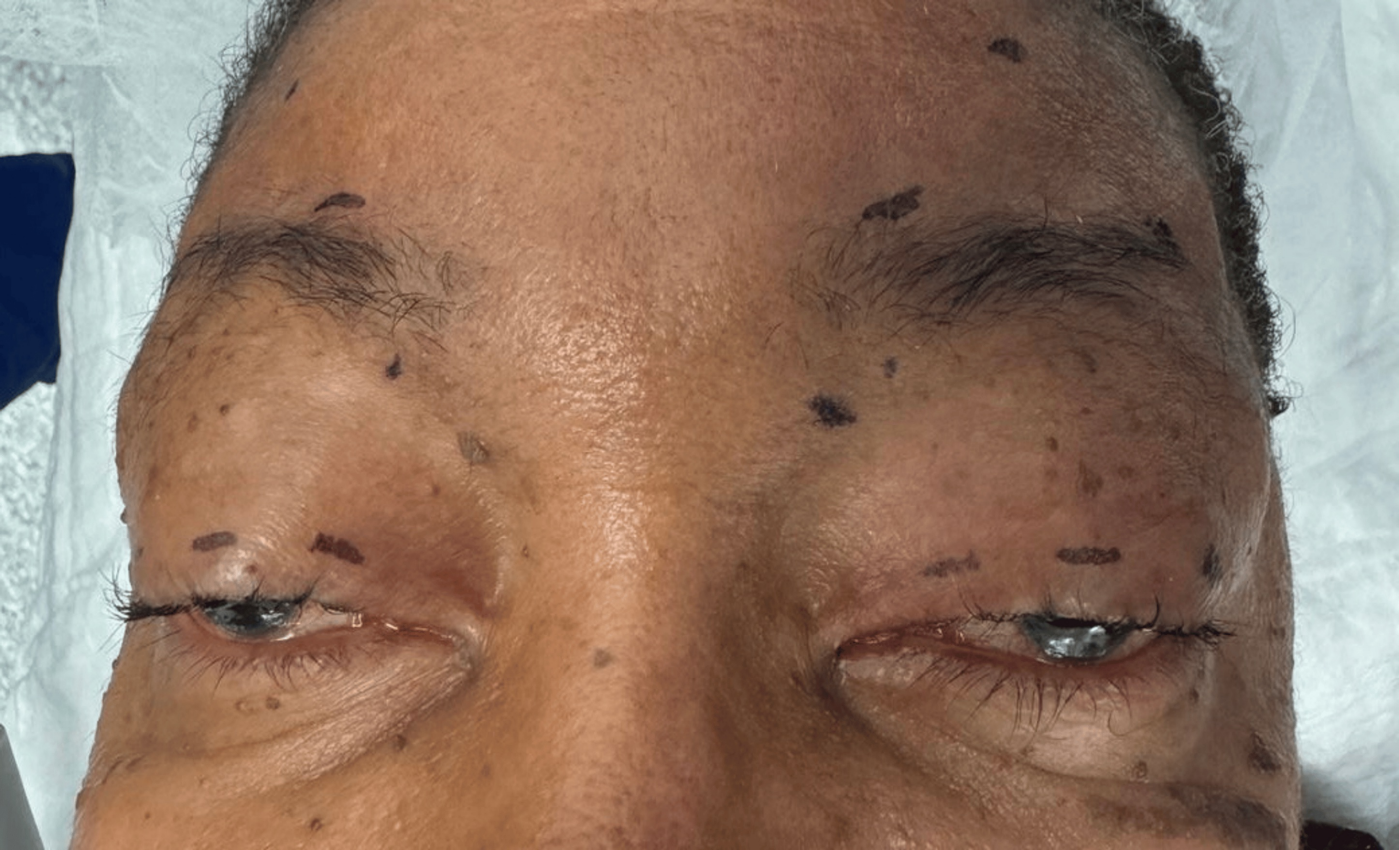
Surgical markings for brow suspension procedure

The procedure involved making incisions in the upper eyelid and along the brow (Figure 4). A silicone sling, chosen for its flexibility and durability, was threaded between the eyelid and the brow. This sling anchored the eyelid to the frontalis muscle, allowing the patient to lift the eyelids by using the forehead muscles. This method compensated for the muscle weakness, improving both function and appearance. The sling was secured and adjusted to ensure optimal eyelid height and symmetry. 

**Figure 5:**
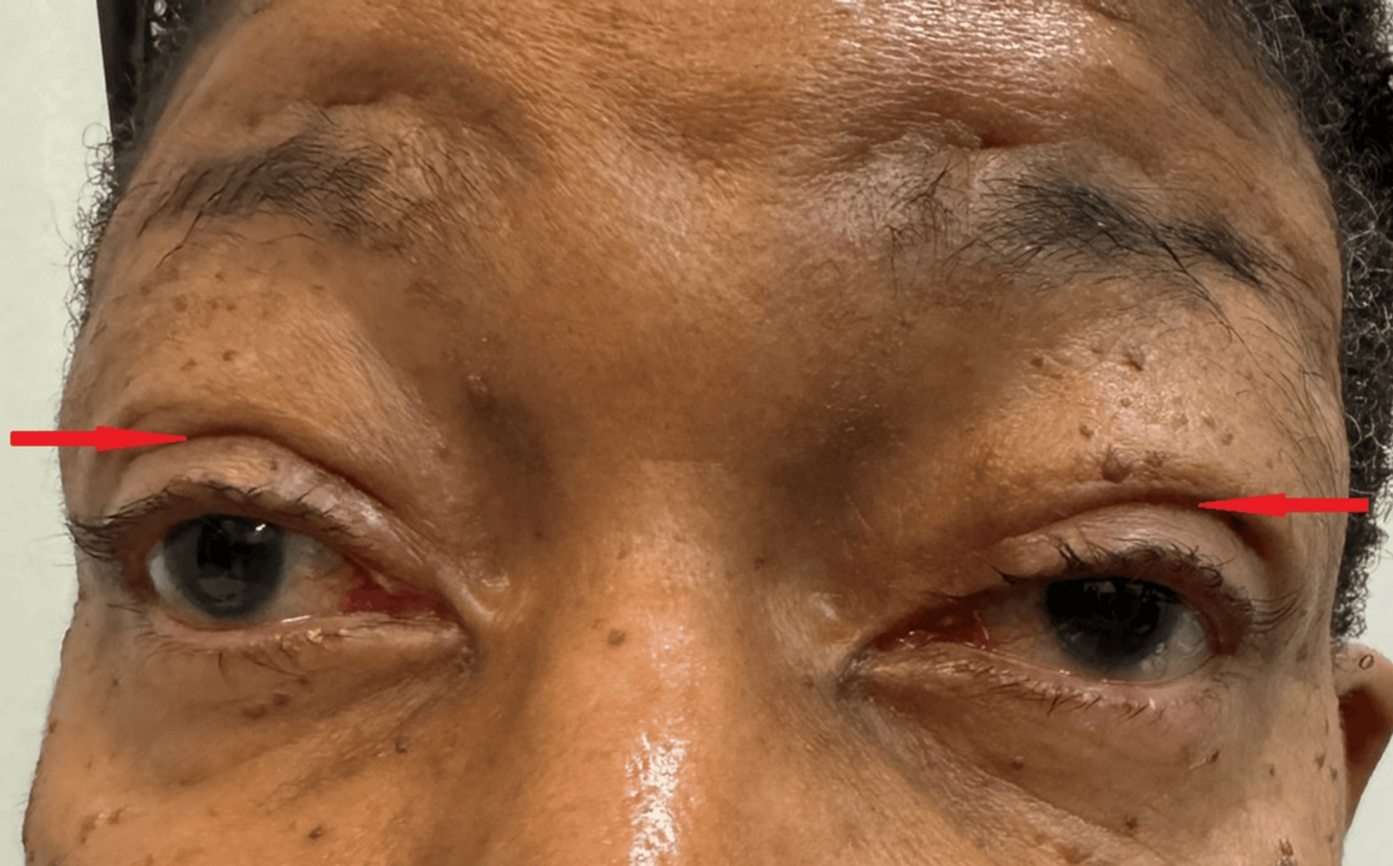
Two months post-ptosis repair surgery, demonstrating significant improvement in ptosis and the reformation of the upper eyelid crease bilaterally (indicated by red arrows)

Postoperatively, the patient's eyelid position significantly improved, restoring a more natural field of vision and reducing the functional limitations caused by ptosis (Figure 5). The surgery was performed as a day case, and the patient was seen the next day for a follow-up. She was prescribed fusidic acid ointment for the incisions twice daily, gatifloxacin eye drops four times daily, and polyacrylic acid 0.2% ointment for nighttime use in both eyes.

## Discussion

CPEO is a rare mitochondrial myopathy characterized by the gradual, symmetric weakening of extraocular muscles, leading to ptosis and ophthalmoplegia. While CPEO can manifest at any age, it typically presents in children and young adults as Kaerns-Sayre syndrome (KSS) [4]. Most cases are sporadic, though approximately one-third are familial [2]. Familial cases follow different inheritance patterns, including autosomal dominant, autosomal recessive, and mitochondrial inheritance, reflecting the genetic diversity of the disorder [1,5]. In this case, the patient admitted to having a sister with ptosis but stated her sister was not diagnosed with CPEO to her knowledge.

The majority of CPEO cases (up to 60%) result from large-scale deletions in mitochondrial DNA (mtDNA), ranging from 1.3 to 9.1 kilobases, and these deletions account for most sporadic cases [1]. A smaller proportion, around 15%, carry point mutations, such as the m.3243A>G mutation in the mitochondrial DNA MT-TL1 gene, which encodes mitochondrial tRNALeu(UUR). This mutation is associated with mitochondrial myopathy, encephalomyopathy, lactic acidosis, and stroke-like episodes (MELAS) [6]. Additionally, autosomal forms of CPEO can result from mutations in nuclear genes essential for the maintenance of mitochondrial DNA [7].

This patient presented with a 36-year history of bilateral progressive ptosis and ophthalmoplegia, absent diplopia, and spared pupils, consistent with CPEO. The slow onset and chronic progression, without periods of exacerbation or remission, are characteristic features of this condition. Given this clinical presentation, various differential diagnoses were considered and excluded.

Kearns-Sayre syndrome (KSS) was ruled out due to the absence of retinal pigmentary degeneration, no nyctalopia, and the late onset of symptoms (after the age of 20). KSS typically manifests earlier and includes additional systemic features such as heart block, deafness, and cerebellar ataxia [8]. Orbital myositis was excluded based on the lack of eye pain, diplopia, proptosis, and chemosis.

Autoimmune myasthenia gravis, which can present with fluctuating ptosis and ophthalmoplegia, was considered unlikely due to the patient’s chronic symptomatology, which did not display the variability seen in myasthenia gravis. Furthermore, there were no associated symptoms like proximal muscle weakness, dysphagia, dysarthria, or abnormalities on electromyography, which are typically seen in myasthenia gravis [9]. The absence of eyelid retraction, proptosis, conjunctival injection, and chemosis, as well as the lack of signs of abnormal thyroid hormone levels, ruled out thyroid eye disease.

Other potential causes of ocular myopathy, such as myotonic dystrophy, oculopharyngeal muscular dystrophy, oculopharyngodistal myopathy, and congenital cranial dysinnervation disorders, were also excluded. For instance, myotonic dystrophy is often associated with systemic symptoms, including distal muscle weakness and myotonia, none of which were present in this patient [10].

## Conclusions

Based on the patient's long-standing bilateral ptosis and ophthalmoplegia without diplopia or pupillary involvement, a clinical diagnosis of isolated CPEO was made. Although further diagnostic testing such as muscle biopsy or genetic analysis would be needed to confirm the diagnosis, the clinical presentation strongly supports sporadic CPEO. The management significantly improved her quality of life, underscoring the importance of recognizing CPEO in patients with chronic ptosis and ophthalmoplegia, even in the absence of genetic or family history. This case is particularly significant; to our knowledge, this is the first reported instance of CPEO in Trinidad and Tobago, highlighting the need for awareness of rare mitochondrial disorders. It also shows that patients can benefit from international collaboration in circumstances where local experience is unavailable. 
